# Sphingomyelin phosphodiesterase-1 (SMPD1) coding variants do not contribute to low levels of high-density lipoprotein cholesterol

**DOI:** 10.1186/1471-2350-8-79

**Published:** 2007-12-18

**Authors:** Zari Dastani, Isabelle L Ruel, James C Engert, Jacques Genest, Michel Marcil

**Affiliations:** 1 From the Cardiovascular Research Laboratories, Division of Cardiology, McGill University Health Centre/Royal Victoria Hospital, Montréal, Québec H3A 1A1, Canada

## Abstract

**Background:**

Niemann-Pick disease type A and B is caused by a deficiency of acid sphingomyelinase due to mutations in the sphingomyelin phosphodiesterase-1 (*SMPD1*) gene. In Niemann-Pick patients, *SMPD1 *gene defects are reported to be associated with a severe reduction in plasma high-density lipoprotein (HDL) cholesterol.

**Methods:**

Two common coding polymorphisms in the *SMPD1 *gene, the G1522A (G508R) and a hexanucleotide repeat sequence within the signal peptide region, were investigated in 118 unrelated subjects of French Canadian descent with low plasma levels of HDL-cholesterol (< 5^th ^percentile for age and gender-matched subjects). Control subjects (n = 230) had an HDL-cholesterol level > the 25^th ^percentile.

**Results:**

For G1522A the frequency of the G and A alleles were 75.2% and 24.8% respectively in controls, compared to 78.6% and 21.4% in subjects with low HDL-cholesterol (*p *= 0.317). The frequency of 6 and 7 hexanucleotide repeats was 46.2% and 46.6% respectively in controls, compared to 45.6% and 49.1% in subjects with low HDL-cholesterol (*p *= 0.619). Ten different haplotypes were observed in cases and controls. Overall haplotype frequencies in cases and controls were not significantly different.

**Conclusion:**

These results suggest that the two common coding variants at the *SMPD1 *gene locus are not associated with low HDL-cholesterol levels in the French Canadian population.

## Background

A low plasma level of high-density lipoprotein (HDL) cholesterol is defined as a cardiovascular risk factor and is part of the assessment of global cardiovascular risk stratification [1]. Therapeutic goals set for the prevention of cardiovascular disease include targets for low density lipoprotein (LDL) cholesterol, non-HDL-cholesterol [2], and the total cholesterol to HDL-cholesterol ratio [3]. However, a goal for an absolute HDL-cholesterol value is still a matter of controversy as the current therapeutic approaches are limited in their ability to raise HDL-cholesterol [4]. In the majority of cases, a low HDL-cholesterol is secondary to increased hepatic secretion of apolipoprotein B-containing lipoproteins and triglycerides [5]. Some cases of low HDL-cholesterol are due to genetic defects in HDL-associated protein apolipoprotein A-I, modifying enzymes (hepatic lipase, lipoprotein lipase, cholesteryl ester transfer protein, lecithin:cholesterol acyl transferase) and the ATP binding cassette A1 transporter [6].

Niemann-Pick disease type A and B is caused by a deficiency of the enzyme acid sphingomyelinase coded by *SMPD1 *gene. *SMPD1 *gene defects are reported to be associated with a severe reduction in plasma HDL-cholesterol [7]. In the search for genes causing disorders of HDL-cholesterol, we examined extended (3 or more generations) kindred of French Canadian descent to identify Mendelian traits. Using this approach, we have previously reported that compound heterozygosity in the *SMPD1 *gene is associated with decreased activity of acid sphingomyelinase and low HDL-cholesterol [8]. Furthermore, a decreased activity of lysosomal and secreted acid sphingomyelinase is believed to cause low HDL-cholesterol in part by decreased activation of lecithin:cholesterol acyltransferase (LCAT) and impaired formation of cholesteryl ester-enriched HDL particles [9]. The *SMPD1 *gene is located on chromosome 11p15.1-p15.4, is comprised of 6 exons, and encodes a cDNA of 2.5 kb. The acid sphingomyelinase protein [10] consists of 631 amino acids and exists in at least three protein isoforms. The protein also contains a signal peptide, a saposin domain that is common in lysosome-targeted proteins, and a large metallophosphoesterase domain. Although rare mutations in the *SMPD1 *gene can impair the function of acid sphingomyelinase and result in Niemann-Pick disease type A or B, as well as in low HDL-cholesterol, it is not known whether common amino acid change variants in *SMPD1 *can modulate HDL-cholesterol levels within a population. The objective of this study was to investigate, in a population selected for HDL-cholesterol levels, associations between known common amino acid variants of the *SMPD1 *gene and low plasma levels of HDL-cholesterol in subjects of French Canadian descent. We focused on frequent (> 5% in the general population) polymorphisms that affect the coding sequence of SMPD1.

## Methods

### Subject characteristics

A total of 348 unrelated subjects of French Canadian origin (118 with low HDL-cholesterol levels and 230 control subjects) were examined at the McGill University Health Centre. Low HDL-cholesterol levels were defined as those less than the 5^th ^percentile (age and gender-matched), based on the Lipid Research Clinics Population Studies Data Book [11]. Subjects with low HDL-cholesterol had no known cause of HDL deficiency (severe hypertriglyceridemia defined as plasma triglycerides > 10 mmol/L, cellular phospholipid or cholesterol efflux defect or previously known mutations in genes associated with HDL deficiency). The control group was of same origin and chosen based on HDL-cholesterol levels > 25^th ^percentile, matched for age and gender. Demographic and clinical information, medications, blood pressure, and lipoprotein profiles were determined on all participating subjects. Hypertension was defined as a blood pressure ≥ 130/85 mmHg. Coronary artery disease (CAD) was present when angiographically documented or patient had a past history of acute myocardial infarction. Consent was obtained for the plasma sampling and DNA isolation. The research protocol was reviewed and approved by the Research Ethics Board of the McGill University Health Centre (REB No. BMA 05-006).

### Measurement of plasma lipids and lipoprotein

The lipid lowering agents were withdrawn in all study subjects for at least four weeks before measurement of the lipid profile. Insulin and oral hypoglycemic agents were maintained in diabetic patients. Plasma was isolated in all study subjects, after a 12-hour fast, in EDTA-containing tubes. Lipids and lipoproteins were measured using standardized techniques and the LDL-cholesterol was calculated according to the Friedewald formula, unless triglyceride levels were > 4.5 mmol/L [12,13].

### DNA analysis

DNA was isolated from the buffy coat obtained after centrifugation of whole blood. Two previously reported common polymorphisms of the *SMPD1 *gene in Niemann-Pick disease type A and B [14,15] were examined. The G→A substitution at position c.1522 located in exon 6 of the *SMPD1 *gene, predicting a substitution of arginine (R) for a glycine (G) at residue 508 (G508R) (rs1050239) was detected by polymerase chain reaction followed by digestion with the restriction enzyme *MspI *(New England Biolabs, MA, USA). The hexanucleotide repeat polymorphism at the start position c.103 (genomic position: 6368507) [10] was located in exon 1 of the *SMPD1 *gene and detected using the sense primer 5'-GTCAGCCGACTACAGAGAAG-3' and the antisense primer 5'-GGCATCTACAATCCATCACT-3'. The antisense primer was radiolabeled at the 5' end with T4 polynucleotide kinase and [γ-^32^P] ATP (PerkinElmer, MA, USA) by standard procedures. Polymerase chain reaction products were resolved on a 6% polyacrylamide denaturating gel.

### Data analysis

The data was analyzed by examining allele frequencies in subjects with low HDL-cholesterol versus controls. The DeFinetti program [16] was employed to test the deviation from Hardy-Weinberg equilibrium and also to compare the frequency of the SNPs between cases and controls. For the G1522A SNP, HDL-cholesterol levels were compared between homozygotes of the common allele and both heterozygotes and homozygotes for the rare allele pooled together. Deviation from Hardy-Weinberg equilibrium for the hexanucleotide repeat polymorphism was tested by PEDSTATS version 0.6.5. The CLUMP program version 2.3 was used to assess the significance of the same marker between cases and controls, by using 1000 simulations in a Monte Carlo approach [17].

Haplotype frequencies (containing both polymorphisms) were estimated for cases and controls, using PHASE version 2.02 [18].

Power calculations using the Genetic Power Calculator [19] demonstrated that we would have > 80% power with our given sample size (controls = 230, cases = 118) to detect a SNP that accounts for 0.02 or more of the variance in HDL-cholesterol. This assumes that we are directly testing a causitive variant with an allele frequency of 23% with a type I error rate α = 0.05.

Statistical analyses were performed with the SAS package version 8 (SAS Institute Inc., NC, USA) and SigmaStat version 2.0 (Jandel Corporation, San Rafael, CA, USA). A χ^2 ^analysis (GraphPad InStat, CA, USA) was performed with respect to allele frequencies in each of the HDL-cholesterol groups. Age, body mass index (BMI), and all lipid parameters in both groups were treated as continuous variables. Comparisons were made through generalized linear model procedures (Proc GLM) followed by Duncan's post hoc test. All *p*-values < 0.05 were considered significant.

## Results

We analyzed a total of 348 subjects from a pool of control subjects and patients with premature CAD. The selection criterion was an HDL-cholesterol < 5^th ^percentile for cases (n = 118), and an HDL-cholesterol > 25^th ^percentile for controls (n = 230). Mean ages were 50 ± 10 and 50 ± 9 years for control and case groups, respectively. Additional demographic and biochemical characteristics are shown in Table 1. Subjects with a low HDL-cholesterol had a higher BMI, were more likely to have type II diabetes, hypertension, CAD and a family history of CAD. These correlates of low HDL-cholesterol have been previously well established [20]. The low HDL-cholesterol group had an HDL-cholesterol of 0.67 ± 0.13 mmol/L and the control group had a mean HDL-cholesterol of 1.35 ± 0.33 (*p *< 0.001). Plasma triglyceride levels were higher in the low HDL-cholesterol group than in the controls (3.95 ± 3.35 mmol/L vs. 1.63 ± 0.89 mmol/L, *p *< 0.001).

**Table 1:** Baseline characteristics of low HDL-C and control subjects

	**Control subjects (n = 230)**	**Low HDL-C subjects (n = 118)**	t test p*
Age (y)	50 ± 9	50 ± 10	0.93
Gender (M/F)	150/80	85/33	0.20
BMI (kg/m^2^)	26.3 ± 4.5	28.1 ± 4.9	**0.001**
DM (%)	10.4	21.2	**0.01**
HTN (%)	20.4	45.8	<**0.001**
CAD (%)	49.8	72.3	<**0.001**
FH of CAD (%)	61.3	72.6	**0.04**
TG (mmol/L)	1.63 ± 0.89	3.95 ± 3.35	<**0.001**
T Chol (mmol/L)	5.73 ± 1.41	5.98 ± 2.20	0.26
HDL-C (mmol/L)	1.35 ± 0.33	0.67 ± 0.13	<**0.001**
LDL-C (mmol/L)	3.70 ± 1.33	3.50 ± 1.61	0.27

BMI, body mass index; DM, diabetes mellitus; HTN, hypertension; CAD, coronary artery disease; FH, familial history; TG, plasma triglycerides; T Chol, total plasma  cholesterol; HDL-C, plasma high-density lipoprotein cholesterol; LDL-C, plasma low-density cholesterol.

* significant *p*-value bolded.

We examined two polymorphisms at the *SMPD1 *gene locus: G1522A (G508R) and a hexanucleotide repeat sequence CTGG (TC)(GT). From our 348 subjects analyzed, 230 controls and 117 cases were successfully genotyped. The prevalence of the G508R variant in cases and controls is shown in Table 2. The presence of the G allele was seen in 75.2% of controls and 78.6% of subjects with low HDL-cholesterol (OR = 0.82; *p *= 0.317). The G allele has been reported at a frequency of 0.85 in an European population [21]. We also separately analyzed the association of this variant between patients with and without CAD and no significant difference was confirmed (*p *= 0.06, data not shown). Genotype frequencies for the GG, GA and AA classes did not differ between subjects with low HDL-cholesterol and controls (Table 2). We examined the association of age, gender, BMI, diabetes mellitus, hypertension, plasma triglycerides, CAD, familial history of CAD, total plasma cholesterol, plasma LDL-cholesterol, between subjects with the GG genotype and subjects with either the AG or AA genotypes in cases and controls (Table 3). We found significant associations between the genotypic classes with familial history of CAD (*p *= 0.0003), total cholesterol (*p *= 0.03) and LDL-cholesterol (*p *= 0.02) in controls. In addition, we found significant associations between the genotypic classes with total cholesterol (*p *= 0.009) in the low HDL-cholesterol subjects. We did not find any significant difference in the prevalence of the GG or AG + AA genotypes with the presence of diabetes. The analysis was also carried out separately between each genotypic class (GG, AG and AA), and the results were similar as those presented in Table 3.

**Table 2:** Genotype distribution and allele frequency of the G1522A variant in the SMPD1 gene in control and low HDL-cholesterol subjects.

**Genotype or allele**	**Control subjects (n)**	**Low HDL-cholesterol subjects (n)**	**χ^2^, two-sided *p*-value and O.R.**
GG	56.1% (129)	64.1% (75)	0.152^†^
AG	38.3% (88)	29.1% (34)	0.72 (0.45–1.13)^‡^
AA	5.6% (13)	6.8% (8)	
G	75.2%	78.6%	0.317
A	24.8%	21.4%	0.82 (0.57–1.20)^‡^

O.R., odd ratio

^† ^AG and AA genotypes where pooled for statistical analysis

^‡ ^95% confidence interval for O.R.

**Table 3:** Comparison of biochemical data of the low HDL-C and control subjects between different groups of G1522A genotypes.

	**Control subjects**		t test p *	**Low HDL-C subjects**		t test p *
				
	**GG**	**AG + AA**		**GG**	**AG + AA**	
n	129	101		75	42	
Age (y)	50 ± 10	50 ± 8	0.5	49 ± 9	51 ± 11	0.4
Gender (M/F)	81/48	69/32	0.3	52/23	32/10	0.4
BMI (kg/m^2^)	26.4 ± 4.6	26.3 ± 4.4	0.9	28.3 ± 5.2	27.7 ± 4.5	0.5
DM (%)	10.5	10.9	0.9	20.3	19.0	0.9
HTN (%)	19.3	22.8	0.5	51.3	33.3	0.06
CAD (%)	45.9	56.0	0.1	75.4	65.8	0.3
FH of CAD (%)	51.7	75.8	**0.0003**	75.0	67.5	0.4
TG (mmol/L)	1.60 ± 0.89	1.70 ± 0.89	0.4	4.34 ± 3.89	3.19 ± 1.90	0.07
T Chol (mmol/L)	5.55 ± 1.19	5.96 ± 1.62	**0.03**	6.33 ± 2.39	5.25 ± 1.56	**0.009**
HDL-C (mmol/L)	1.34 ± 0.31	1.33 ± 0.33	0.8	0.67 ± 0.13	0.67 ± 0.13	0.9
LDL-C (mmol/L)	3.51 ± 1.10	3.93 ± 1.54	**0.02**	3.71 ± 1.74	3.17 ± 1.34	0.09

BMI, body mass index; DM, diabetes mellitus; HTN, hypertension; CAD, coronary artery disease; FH, familial history; TG, plasma triglycerides; T Chol, total plasma cholesterol; HDL-C, plasma high-density lipoprotein cholesterol; LDL-C, plasma low-density cholesterol.

* significant *p*-value bolded.

The second polymorphism consisted of a unique hexanucleotide sequence CTGG(TC)(GT) located within the signal peptide region of the acid sphingomyelinase (corresponding to the hydrophobic sequence LVLALALALALA). The genotype distribution of the hexamer polymorphism was examined and the most frequent allele was the 6 and 7 repeats (respectively 46% and 47% of the control group) (Table 4). We identified 9 genotypes in our study population with the most prevalent being 6/7, 7/7 and 6/6. There was no significant difference in the genotype or allele frequencies between low HDL-cholesterol subjects and controls. We used the CLUMP program to confirm these results with a *p*-value of 0.6 after a 1000-simulation analysis. We examined the most frequent genotypes with respect to age, gender, BMI, diabetes mellitus, CAD, family history of CAD, triglycerides, total cholesterol and LDL-cholesterol levels and we found significant differences between the subgroups of subjects with 6/6, 6/7, 7/7 for hypertension (*p *= 0.04) and triglycerides (*p *= 0.005) in low HDL-cholesterol subjects only (Table 5).

**Table 4:** Genotype distribution and allele frequency of the hexanucleotide repeat polymorphism in the SMPD1 gene in low HDL-C and control subjects

**Genotype or allele**	**Control subjects (%)**	**Low HDL-C subjects (%)**	***p***
5/5	0.4	0	0.527
5/6	6.3	2.6	
5/7	5.4	7.0	
6/6	20.3	25.5	
6/7	45.6	37.7	
7/7	20.3	26.3	
7/8	0.4	0	
7/9	0.9	0.9	
7/10	0.4	0	

5	6.3	4.8	0.619
6	46.2	45.6	
7	46.6	49.1	
8	0.2	0	
9	0.5	0.5	
10	0.2	0	

**Table 5:** Comparison of biochemical data of the low HDL-C and control subjects between the most prevalent genotypes of the hexanucleotide repeat polymorphism in the SMPD1 gene.

	**Control subjects (n = 230)**			**t test *p***	**Low HDL-C subjects (n = 118)**			t test p*
				
	**6/6**	**6/7**	**7/7**		**6/6**	**6/7**	**7/7**	
n	45	101	45		29	43	30	
Age (y)	50 ± 8	50 ± 9	49 ± 9	0.79	48 ± 10	51 ± 10	49 ± 10	0.51
Gender (M/F)	30/15	68/33	29/16	0.94	22/7	29/14	20/10	0.69
BMI (kg/m^2^)	26.1 ± 5.1	26.4 ± 4.0	26.0 ± 4.1	0.86	27.9 ± 4.2	27.8 ± 5.1	29.2 ± 5.6	0.45
DM (%)	15.0	55.0	30.0	0.57	19.0	42.9	38.1	0.47
HTN (%)	23.1	61.5	15.4	0.35	22.2	35.6	42.2^†^	**0.04**
CAD (%)	26.1	51.1	22.8	0.77	23.9	45.1	31.0	0.16
FH of CAD (%)	28.3	53.3	18.3	0.05	23.6	43.1	33.3	0.15
TG (mmol/L)	1.60 ± 0.68	1.56 ± 0.85	1.68 ± 0.88	0.71	2.73 ± 1.38	4.94 ± 3.65^†^	3.57 ± 2.58	**0.005**
T Chol (mmol/L)	6.09 ± 1.45	5.61 ± 1.52	5.82 ± 1.32	0.19	5.56 ± 2.21	6.12 ± 1.77	6.03 ± 2.06	0.48
HDL-C (mmol/L)	1.36 ± 0.34	1.33 ± 0.31	1.31 ± 0.28	0.80	0.66 ± 0.11	0.67 ± 0.13	0.69 ± 0.10	0.55
LDL-C (mmol/L)	3.96 ± 1.37	3.60 ± 1.34	3.79 ± 1.39	0.32	3.60 ± 2.08	3.38 ± 1.34	3.68 ± 1.62	0.74

BMI, body mass index; DM, diabetes mellitus; HTN, hypertension; CAD, coronary artery disease; FH, familial history; TG, plasma triglycerides; T Chol, plasma cholesterol; HDL-C, plasma high-density lipoprotein cholesterol; LDL-C, plasma low density lipoprotein cholesterol.

^†^significantly different from genotype 6/6.

* significant *p *value bolded.

We used the PHASE program to reconstruct haplotypes in cases and controls. Substantial linkage disequilibrium was observed as the A allele of G1522A was seen almost exclusively with the hexanucleatide repeat of "6" (Table 6). Overall haplotype frequencies between cases and controls were not significantly different (*p *= 0.5) (Table 6).

**Table 6:** Estimation of the haplotype frequency distribution of the G1522A variant and hexanucleotide repeat polymorphism in the SMPD1 gene in low HDL-C and control subjects.

**Haplotype**	**Total (%)**	**Controls subjects (%)**	**Low HDL-C subjects (%)**
5 G	5.5	5.8	4.7
5 A	0.4	0.5	0.1
6 G	23.3	22.7	24.5
6 A	22.4	23.1	21.1
7 G	46.9	45.8	48.9
7 A	0.8	1.2	0.2
8 G	0.2	0.2	0.0
9 G	0.4	0.4	0.4
10 G	0.2	0.2	0.0

*p*-value for testing H_0_: cases ~ controls = 0.5.

## Discussion

The present report suggests that common genetic variability at the *SMPD1 *gene locus does not contribute significantly to HDL-cholesterol levels in a French Canadian population. Rare mutations at the *SMPD1 *gene can cause Niemann-Pick disease type A or B, which can differ in degrees of neurological impairment. Mutations for both types A and B are distributed throughout the *SMPD1 *gene and the structure-function relationship between mutations and disease states is not fully understood. We and others have previously reported that patients with Niemann-Pick disease type A/B have low plasma levels of HDL-cholesterol [7,8,22]. More recently, we have shown that cellular cholesterol processing is abnormal in fibroblasts with *SMPD1 *mutations [9]. Despite an abnormal lysosomal transport of cholesterol and sphingomyelin, cellular cholesterol efflux onto apolipoprotein A-I does not appear to be the rate-limiting step in generating nascent HDL particles. Instead, our data suggests that abnormal composition of nascent HDL particles leads to abnormal LCAT activity and decreased cholesterol esterification when the protein product of the *SMPD1 *gene, acid sphingomyelinase, is defective [9]. It has been previously reported that reconstituted HDL particles using proteoliposomes with an increasing ratio of sphingomyelin to phosphatidylcholine inhibits LCAT activity and cholesteryl ester formation [23-25]. This leads to an inability of HDL particles to mature into spherical HDL_3 _particles. In turn, current evidence points to an increased catabolism of these nascent, cholesteryl ester-poor HDL particles by the kidney [26].

In a previous report, we have shown that rare mutations of the *SMPD1 *gene leads to reduced activity of acid sphingomyelinase and is associated with a low HDL-cholesterol. In addition, the mutations segregate within families with a gene dosage effect. This gene-dosage effect was shown in HDL-cholesterol levels in homozygotes and compound heterozygotes [8]. Here, we have found that the prevalence of the G1522A substitution (G508R) was not significantly different in subjects with a low HDL-cholesterol, compared with controls. We used the PolyPhen program [27] to determine the predicted impact of individual variants on *SMPD1 *function and this variant was predicted to be benign.

Moreover, the presence of the 6 and 7 hexanucleotide repeats as well as the 10 different haplotypes in cases and controls were not significantly different. In a previous report, the 5 separate alleles, corresponding to 9, 7, 6, 5 and 4 hexanucleotide repeats were unrelated to Niemann-Pick disease [15]. Corresponding allele frequencies of 0.5%, 12.4%, 50.4%, 34.9% and 1.8% were found in that study, generating 9 different genotypes [15].

Some significant associations were found between *SMPD1 *genotypic classes and characteristics of the cases and control subjects. For example, in the control group, carriers of the 1522A had a significant increase in family history of CAD, plasma LDL-cholesterol and total cholesterol levels. However, the 1522A allele was associated with lower total plasma cholesterol concentrations in the cases. Given the lack of consistency of these results between the control and low HDL-cholesterol groups, and the relative statistical weakness of these associations (not significant or only marginally significant when Bonferroni corrections are applied for multiple testing), the clinical relevance of these findings is uncertain and are probably the result of multiple statistical tests.

This study is limited by the relatively small number of subjects (n = 348). However, we did have greater than 80% power to detect a genetic variant that accounts for as little as 2% of the variance of HDL-cholesterol. In addition, we used arbitrary cut-points of an HDL-cholesterol < 5^th ^percentile and > 25^th ^percentile for cases and controls, respectively. These data should still be confirmed in a large-scale study.

## Conclusion

Our data suggest that while rare mutations at the *SMPD1 *locus can cause Niemann-Pick disease types A and B and the concomitant low HDL-cholesterol, the two common coding non-synonymous variants that we examined at this locus do not appear to influence HDL-cholesterol levels to any great extent. Forty-five mutations in *SMPD1 *gene causing different forms of Niemann-Pick disease type A and B have been described [28]. Since the incidence of Niemann-Pick disease type B is difficult to estimate due to the lack of enzyme testing in clinic, variability in symptoms and the lack of knowledge of Niemann-Pick disease type B by treating physicians, many patients remain undiagnosed [28]. It remains to be determined if variations in the *SMPD1 *gene, affecting the activity of acid sphingomyelinase, might contribute to the modulation of HDL-cholesterol levels in the general population. This study did not examine rare mutations and thus carrier status for Niemann-Pick disease type B was not ruled out in either group. However, Niemann-Pick disease type B should not be common enough to influence our findings.

## Abbreviations

BMI, body mass index; CAD, coronary artery disease; HDL, high-density lipoprotein; LCAT, lecithin:cholesterol acyltransferase; LDL, low density lipoprotein; SMPD1, sphingomyelin phosphodiesterase-1.

## Competing interests

The author(s) declare that they have no competing interests.

## Authors' contributions

ZD and JCE carried out the analysis of data, participated in the design of the study and the writing of the paper; ILR and ZD carried out the genotyping of the samples; ILR participated in the data analysis and the writing of the paper; MM participated in the patient collection and characterization, the design of the study, the genotyping of the samples, the data analysis and the writing of the paper; JG carried out the clinical examination and collection of the patients, the design of the study and participated in the writing of the paper. All the authors read and approved the final manuscript.

## Pre-publication history

The pre-publication history for this paper can be accessed here:


